# Trends in telemedicine utilization for mental illness during the COVID-19 pandemic: an analysis of a nationwide database in Korea

**DOI:** 10.1186/s12888-023-05258-x

**Published:** 2023-10-24

**Authors:** Kyoung Hoon Kim, Sang Min Lee, Minha Hong, Kyu-Man Han, Jong-Woo Paik

**Affiliations:** 1 Department of Health Administration, College of Nursing and Health, Kongju National University, Gongju, Republic of Korea; 2 Department of Psychiatry, Kyung Hee University Hospital, Kyung Hee University College of Medicine, Seoul, Republic of Korea; 3 UNC Neuroscience Center, University of North Carolina, Chapel Hill, NC USA; 4 Department of Psychiatry, Myongji Hospital, Hanyang University College of Medicine, Goyang, Republic of Korea; 5 Department of Psychiatry, Korea University College of Medicine, Seoul, Republic of Korea

**Keywords:** COVID-19 pandemic, Mental illness, Telemedicine utilization

## Abstract

**Background:**

The coronavirus disease 2019 (COVID-19) pandemic has worsened mental health and reduced access to mental health services. During the pandemic, the demand for telemedicine has increased and related laws have been enacted. This study aimed to investigate telemedicine use for cases of major mental illnesses during the COVID-19 pandemic and to compare the characteristics of patients who received telemedicine service with those of patients who received in-person care.

**Methods:**

This population-based, cross-sectional, observational study was based on health insurance claims data, and included 2,749,872 patients who received outpatient treatment for mental illness from February 24, 2020 to June 30, 2022. Logistic regression was performed to assess the relationships between patient characteristics and telemedicine service use. Patients who received telemedicine services were analyzed in subgroups of each mental illness.

**Results:**

During the study period, 80,157 patients (2.9%), with an average age of 63 years, received at least one telemedicine treatment. There was a predominance of women and medical aid recipients. The lowest proportion of telemedicine treatments was for depression (2.1%), and the highest was for dementia (6.7%). The proportion of patients receiving telemedicine in long-term care hospitals was high (22.6%), with the highest odds ratio (OR) (5.84), compared with that in tertiary or general hospitals, followed by that in psychiatric hospitals and clinics. The proportions were high in the departments of internal medicine, neurology, and psychiatry. Patients aged > 80 years received most telemedicine treatment (OR: 1.23) across all diagnoses. Cases of dementia and other mental disorders had higher ORs (2.60 and 2.36, respectively) compared with cases of depression. Except for dementia and behavioral/emotional disorders, hospitalization increased the probability of telemedicine treatment. Comorbidities were positively associated with telemedicine treatment.

**Conclusions:**

Older people and people with other physical illnesses were more likely to use telemedicine treatments temporarily provided during the pandemic. Telemedicine maintained continuity of treatment for patients with dementia and severe mental illnesses. Telemedicine can be useful for filling the medical gaps for vulnerable populations other than those with mild mental illnesses. This aspect should be considered for the future establishment of telemedicine systems.

## Background

Owing to the coronavirus disease 2019 (COVID-19) pandemic, interest and use of telemedicine worldwide has increased and the related laws have changed rapidly [1, 2]. In March 2020, the United States government granted a drastic temporary exemption from regulations and rules on telehealth, and from February 24, 2020, the Korea Ministry of Health and Welfare also temporarily allowed phone consultations and prescriptions through telemedicine [3]. As visiting hospitals has been challenging owing to social distancing and isolation, the demand for telemedicine has increased rapidly [4]. In Korea, telemedicine was prohibited in principle before the outbreak of COVID-19 owing to the relatively easy access to medical care compared to other countries and opposition from stakeholders [5]. Its introduction and use lagged behind that of other countries [6]. However, the changed medical environment because of the COVID-19 pandemic resulted in the government rapidly promoting a plan to support telemedicine services to ensure public safety and protect medical personnel from infectious agents [7].

As a public health measure against the pandemic, social distancing and isolation have been introduced worldwide, which increased loneliness and decreased social support, leading to increased anxiety, depression, and stress in the general population [8]. Although concerns regarding mental health have increased during the pandemic, the psychological impact of COVID-19 on patients with pre-existing mental disorders may be more pronounced [9]. People receiving psychiatric treatment for schizophrenia, bipolar disorder, depression, or anxiety disorders had poor disease control during the pandemic [10]. Most people with mental illnesses must visit outpatient departments regularly for prescriptions. In China, at the beginning of the pandemic, 17.2% of patients with mental disorders stopped receiving medication owing to difficulties in visiting hospitals [11]. According to the World Health Organization, the COVID-19 pandemic has increased the demand for mental health services, and essential mental health services were stopped or disrupted in 93% of countries [12].

The pandemic does not impact populations equally, and those with mental health disorders are vulnerable to these impacts [13]. There are concerns that those most in need may be excluded from access to technology and the internet, and this digital equity issue also involves mental health [14].

In the future, telemedicine treatment will be used more widely in managing mental health issues. Telemedicine is beneficial in terms of patient satisfaction, privacy, and time savings, and is helpful in areas or targets where healthcare access is limited [15]. Moreover, it has been recognized as a promising solution to address gaps in mental health access [16], as it can be used effectively in anxiety, depression, and substance use disorders. Further, it is known to be useful in primary health care settings because it avoids stigma and ensures privacy [17, 18]. Finally, it can provide convenience to patients with special needs, such as young people, minorities, and older adults [18].

Telemedicine use in mental health issues, which have surged during the pandemic, has been proven effective and safe in evaluation and management [19]. A scoping review of tele-mental health use during the COVID-19 pandemic reported that there was more research on general mental health care than that for mental disorders. It emphasized the need for more discussion on telemedicine use for prevention and early diagnosis of mental illness [20]. Previous studies have investigated the factors that are necessary for the implementation of telemedicine and the effect of improving mental health during the COVID-19 pandemic [21, 22]. However, to our knowledge, there has been no study to examine for which psychiatric diseases telemedicine is more commonly used.

Therefore, this study aimed to investigate the status of telemedicine in the mental health area across the country during the COVID-19 pandemic. Additionally, we hypothesized that people with mild mental illness would be more likely to receive telemedicine service because they would have better access to telemedicine services and could more easily adapt to the transition to telemedicine. People with mild mental illness were defined as patients with no hospitalization experience and a low incidence of comorbidities. Our ultimate aim was to use the results of this study to improve telemedicine policies.

## Methods

### Study design and data source

This population-based cross-sectional study identified the status of telemedicine treatment for patients with mental illness during the COVID-19 pandemic and compared the characteristics of patients who received telemedicine and those who received in-person therapies.

As telemedicine was allowed temporarily from February 24, 2020, the National Health Insurance Claim Database (NHICD) was utilized for medical expense reviews from February 24, 2020 to June 30, 2022. Korea has a single health insurance system, and the Health Insurance Review and Evaluation Service collects and manages patient medical information. The NHICD contains data on demographic characteristics, diagnoses, and the healthcare facilities used [23]. The authors assert that all procedures contributing to this work comply with the ethical standards of the relevant national and institutional committees on human experimentation and with the Helsinki Declaration of 1975, as revised in 2008. All procedures involving human subjects/patients were approved by the Institutional Review Board (IRB) of Myongji Hospital (IRB NO.2022-04-029). The IRB of Myongji Hospital waived the requirement for informed consent because of the public nature of NHICD data, which contain information by ID number but are not identifiable.

### Study population

People with mental illness were defined as patients of any age with a primary F diagnosis code in the International Classification of Disease, 10th version. During the study period, 32,955 healthcare facilities offered outpatient care for people with mental illness, of which 4,167 (12.6%) provided at least one telemedicine treatment. Among patients who received outpatient treatment for mental illness, 2,749,872 people who received telemedicine or in-person treatment at hospitals where telemedicine was available were included in this study. Patients who received telemedicine treatment at least once were classified into the telemedicine group (80,157), and those who only received outpatient treatment were classified into the in-person group (2,669,715).

### Definition of variables

Data on demographic and medical characteristics were collected for comparison. Demographic characteristics included sex, age, and type of medical coverage, using age ranges of < 20, 20–44, 45–64, 65–79, and ≥ 80 years. Medical coverage was classified into health insurance and medical aid.

Medical information included the diagnosis, type of healthcare facility, department, medical history, and the charlson comorbidity index (CCI). The diagnoses were classified into depression (F32–F39); anxiety (F40 and F41); dementia (F00–F03); brain injury; brain dysfunction and other mental disorders due to brain damage and dysfunction and physical disease (F06); sleep disorders (F51); schizophrenia (F20–F29); behavioral and emotional disorders (BEDs) with onset usually in childhood and adolescence (F90–F98); bipolar affective disorder (F30 and F31); and others, based on frequent diseases during the study period. Healthcare facilities were divided into tertiary or general hospitals, long-term care hospitals, psychiatric hospitals, and primary clinics, and the departments were those of psychiatry, neurology, internal medicine, and others. Medical history was defined as the number of hospitalizations and outpatient visits in the past year, by setting the time of receiving a telemedicine service as the index date. The number of outpatient visits was divided into 0, 1–2, 3–5, 6–9, and ≥ 10, including only outpatient visits due to mental illness. CCI, a surrogate variable for the patient’s health status, was also observed for 1 year, based on the index date, and was divided into 0, 1, 2, and ≥ 3 points.

### Statistical analysis

To check the number of telemedicine treatments after February 24, 2020, the number of treatments for mental illness was analyzed monthly.

The characteristics of patients who received telemedicine or only in-person services were analyzed using frequency and percentage for categorical variables and means and standard deviations for continuous variables. The statistical significance of differences in the characteristics between the two groups was analyzed using the Chi-square test and Student’s t-test. Logistic regression analysis was performed to investigate the relationships between patient characteristics and telemedicine service use. Patient characteristics included age, sex, diagnosis, healthcare facility characteristics, and medical history. Additionally, logistic regression was performed for each mental illness, to confirm the characteristics of patients who received telemedicine services, as a subgroup analysis. The reference age was set to 20 years, except for case of patients with dementia where it was set at 20–44 years because dementia did not occur in individuals aged < 20 years.

The SAS Enterprise Guide 7.1. (SAS Institute Inc., Cary, NC, US) was used for data construction and analysis, and statistical significance was set at 5%.

## Results

### General characteristics of patients who received telemedicine and in-person care

Among patients who received outpatient treatment for mental illness, 80,157 (2.9%) received at least one telemedicine service during the study period, and 2,669,715 patients (97.1%) only received in-person services (Table 1). The average age of the patients who received telemedicine services was 63 years, which was higher than that of patients who received in-person services (53.2 years). More female patients (3.2%) received telemedicine services than male patients (2.5%), and medical aid recipients received more telemedicine services than patients with health insurance. Regarding the number of outpatient visits, the diagnostic frequency, from highest to lowest, was depression, anxiety disorders, dementia, other mental disorders, sleep disorders, schizophrenia, behavioral and emotional disorders, bipolar disorders, and others. Among the included patients, 6.7%, 4.4%, 3.9%, and 3.6% of those with dementia, schizophrenia, bipolar affective disorder, and other mental disorders, respectively, received telemedicine. The highest proportion of telemedicine treatments (22.6%) was reported among patients who received treatment in long-term care hospitals, followed by those who received treatment in hospitals and psychiatric hospitals. Telemedicine services were provided by the departments of internal medicine (5.2%), neurology (2.9%), and psychiatry (2.62%). Based on the time point of receiving a telemedicine service, the higher the number of hospitalizations or outpatient visits within the past year, the higher the proportion of patients who received telemedicine service (P < 0.001) (Table 1). Among patients with prior hospitalizations, 4.9% received telemedicine services, compared with 2.9% of patients who had not been hospitalized. Additionally, 0.5% of patients with no prior outpatient visits received a telemedicine service. In comparison, 6.2% of patients who visited outpatient clinics > 10 times received telemedicine service.

**Table 1:** General characteristics of patients receiving telemedicine or in-person care

Variables	Category	Total	In-person care	Telemedicine	*P*-value
**Total (%)**		2,749,872(100.0)	2,669,715(97.1)	80,157(2.9)	
**Age (years)**		53.5 ± 23.0	53.2 ± 22.9	63.0 ± 22.8	< 0.0001
	< 20	228,177(8.3)	224,133(98.2)	4,044(1.8)	< 0.0001
	20–44	750,163(27.3)	736,153(98.1)	14,010(1.9)	
	45–64	736,739(26.8)	719,649(97.7)	17,090(2.3)	
	65–79	632,820(23.0)	612,459(96.8)	20,361(3.2)	
	≥ 80	401,973(14.6)	377,321(93.9)	24,652(6.1)	
**Sex**	Male	1,079,203(39.2)	1,052,183(97.5)	27,020(2.5)	< 0.0001
	Female	1,670,669(60.8)	1,617,532(96.8)	53,137(3.2)	
**Insurance Type**	Health insurance	2,492,499(90.6)	2,424,681(97.3)	67,818(2.7)	< 0.0001
	Medical aid	257,373(9.4)	245,034(95.2)	12,339(4.8)	
**Type of healthcare facility**	Tertiary/general hospital	884,425(32.2)	864,517(97.8)	19,908(2.3)	< 0.0001
	Hospital	163,873(6.0)	156,478(95.5)	7,395(4.5)	
	Long-term care hospital	13,017(0.5)	10,070(77.4)	2,947(22.6)	
	Psychiatric Hospital	71,617(2.6)	69,073(96.5)	2,544(3.6)	
	Primary clinic	1,616,940(58.8)	1,569,577(97.1)	47,363(2.9)	
**Diagnosis**	Depression	669,974(24.4)	655,913(97.9)	14,061(2.1)	< 0.0001
	Anxiety disorders	521,801(19.0)	511,865(98.1)	9,936(1.9)	
	Dementia	390,706(14.2)	364,586(93.3)	26,120(6.7)	
	Other mental disorders	221,781(8.1)	213,746(96.4)	8,035(3.6)	
	Sleep disorders	215,581(7.8)	210,744(97.8)	4,837(2.2)	
	Schizophrenia	118,513(4.3)	113,316(95.6)	5,197(4.4)	
	BEDs	98,554(3.6)	96,467(97.9)	2,087(2.1)	
	Bipolar disorder	71,066(2.6)	68,306(96.1)	2,760(3.9)	
	Other	441,896(16.1)	434,772(98.4)	7,124(1.6)	
**Department**	Psychiatry	2,107,293(76.6)	2,053,440(97.4)	53,853(2.6)	< 0.0001
	Neurology	301,460(11.0)	292,635(97.1)	8,825(2.9)	
	Internal medicine	179,314(6.5)	169,990(94.8)	9,324(5.2)	
	Other	161,805(5.9)	153,650(95.0)	8,155(5.0)	
**Previous admission**	No	2,662,764(96.8)	2,586,849(97.2)	75,915(2.9)	< 0.0001
	Yes	87,108(3.2)	82,866(95.1)	4,242(4.9)	
**Numbers of previous outpatient care**	0	1,426,569(51.9)	1,419,210(99.5)	7,359(0.5)	< 0.0001
	1–2	288,205(10.5)	276,754(96.0)	11,451(4.0)	
	3–5	291,767(10.6)	275,975(94.6)	15,792(5.4)	
	6–9	245,869(8.9)	231,254(94.1)	14,615(5.9)	
	≥ 10	497,462(18.1)	466,522(93.8)	30,940(6.2)	
**CCI score**	0	1,294,150(47.1)	1,268,898(98.1)	25,252(2.0)	< 0.0001
	1	689,748(25.1)	666,948(96.7)	22,800(3.3)	
	2	373,119(13.6)	357,470(95.8)	15,649(4.2)	
	≥ 3	392,855(14.3)	376,399(95.8)	16,456(4.2)	

Abbreviations: BED, behavioral and emotional disorders with onset usually in childhood and adolescence; CCI, Charlson comorbidity index; other mental disorders, other mental disorders due to brain damage and dysfunction and physical disease

**Fig. 1:**
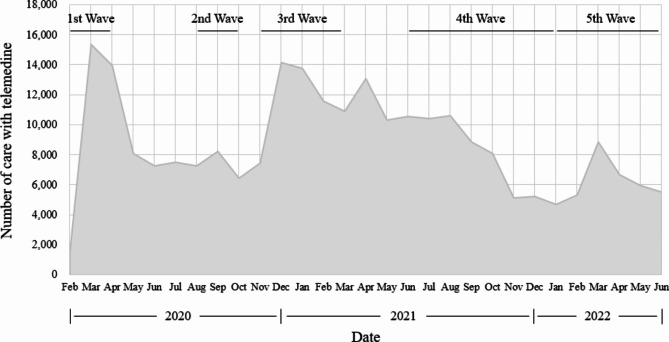
Trends in the numbers of patients with mental illness who received telemedicine services during the COVID-19 pandemic Note) 1st wave: February-March 2020, 2nd wave: August-September 2020, 3rd wave: November 2020-February, 2021, 4th wave: June-December, 2021 5th wave: January-May, 2022.

During the first wave of COVID-19 (February to March 2020), the number of telemedicine treatments was highest in March 2020, shortly after telemedicine was temporarily allowed (Fig. 1). This trend decreased subsequently. However, during the second and third waves, (August to September 2020 and November 2020 to February 2021, respectively), it increased again. During the fourth wave (June to December 2021), the number of telemedicine treatment was maintained and then decreased, and during the fifth wave (January to May 2022), it increased again.

### Associations between general characteristics and telemedicine care

The characteristics of patients who received telemedicine services were analyzed by comparing them with patients who only received in-person services. Compared with patients under 20 years, those aged 20–44, 44–64, and 65–79 years received significantly fewer telemedicine treatment, and the odds ratio (OR) of patients aged ≥ 80 years was 1.23 (95% confidence interval [CI]: 1.17–1.29), indicating that they were more likely to receive telemedicine services (Table 2). The OR between women and men who received telemedicine services was 1.07 (95% CI: 1.06–1.09). Patients treated in long-term care hospitals had the highest OR of receiving telemedicine services, of 5.84 (95% CI: 5.56–6.13), compared with those treated in tertiary and general hospitals, followed by ORs of 2.04 (95% CI: 1.95–2.13) and 1.96 (95% CI: 1.92–1.99) for those treated in mental hospitals and clinics, respectively.

Patients with prior hospitalization had an OR of 1.06 (95% CI: 1.02–1.09) of receiving telemedicine services compared with patients without previous hospitalization. The OR of receiving telemedicine services increased linearly with increased outpatient visits. Compared to patients with a CCI score of 0 points overall, and in each disease group, patients with a CCI score of ≥ 1 point also had higher ORs of receiving telemedicine services. Patients with mental disorders other than depression were more likely to receive telemedicine services. Patients with dementia and other mental disorders had ORs of 2.60 (95% CI: 2.53–2.67) and 2.36 (95% CI: 2.29–2.44), respectively, of receiving telemedicine services, which was higher than those of other diseases.

When analyzed by type of mental illness, adults aged ≥ 80 years received telemedicine services more frequently regardless of the type of illness, compared with patients with depression or anxiety disorders aged < 20 years, or those of other age groups. Patients with sleep disorders, schizophrenia, and bipolar affective disorder aged ≥ 80 years also received more telemedicine services than those aged < 20 years. However, the patients with sleep disorders and schizophrenia did not differ significantly in terms of the likelihood of receiving telemedicine services in other age groups, unlike depression. Among patients with other mental disorders due to brain damage, dysfunction, and physical illness, women, and medical aid recipients were significantly less likely to receive telemedicine services than men and patients with health insurance. A consistent association was found in most psychiatric disorders by the type of healthcare facilities compared to the overall outcome. Nevertheless, patients treated for schizophrenia at clinics had an OR of 0.90 (95% CI: 0.82–0.98) of receiving telemedicine services compared with those treated at tertiary and general hospitals.

Patients with prior hospitalization were more likely to receive telemedicine services for all diagnoses except dementia and BED. The higher the number of outpatient visits, the higher the odds of receiving telemedicine services for all disease types. Compared with patients with a CCI score of 0 points without coexisting diseases, patients with CCI score ≥ 3 points were more likely to have received telemedicine; however, patients with schizophrenia with CCI score ≥ 3 points were an exception, with an OR of 0.86 (95% CI: 0.77–0.96).

**Table 2:** Logistic regression results on the association between patient characteristics and use of telemedicine care

Variables	Category	Total	Depression	Anxiety disorders	Dementia	Other mental disorders	Sleep disorders	Schizophrenia	Behavioral and emotional disorders	Bipolar disorder
		OR (95% CI)	OR (95% CI)	OR (95% CI)	OR (95% CI)	OR (95% CI)	OR (95% CI)	OR (95% CI)	OR (95% CI)	OR (95% CI)
Age (years)	< 20	1.00	1.00	1.00		1.00	1.00	1.00	1.00	1.00
	20–44	0.85 (0.82–0.89)	0.92 (0.92–0.93)	0.92 (0.82–1.03)		1.23 (0.52–2.91)	0.92 (0.64–1.34)	0.80 (0.63–1.03)	0.82 (0.73–0.92)	0.78 (0.65–0.94)
	45–64	0.87 (0.84–0.91)	0.81 (0.81–0.81)	0.92 (0.82–1.03)	1.18 (0.64–2.20)	1.58 (0.69–3.61)	0.98 (0.68–1.42)	0.91 (0.71–1.17)	1.14 (0.83–1.55)	0.75 (0.62–0.90)
	65–79	0.94 (0.90–0.99)	0.86 (0.86–0.86)	0.87 (0.77–0.98)	1.13 (0.61–2.09)	1.49 (0.66–3.40)	0.94 (0.65–1.36)	1.36 (1.05–1.75)	1.66 (0.91–3.02)	1.04 (0.85–1.28)
	≥ 80	1.23 (1.17–1.29)	1.44 (1.44–1.45)	1.56 (1.37–1.78)	1.29 (0.70–2.38)	1.95 (0.86–4.45)	1.62 (1.12–2.36)	2.04 (1.52–2.72)	1.64 (0.72–3.75)	2.02 (1.62–2.51)
Sex	Male	1.00								
	Female	1.07 (1.06–1.09)	1.16 (1.16–1.16)	1.22 (1.17–1.28)	1.02 (0.99–1.05)	0.88 (0.84–0.93)	1.23 (1.16–1.31)	1.09 (1.03–1.16)	1.09 (0.98–1.20)	1.07 (0.99–1.16)
Insurance Type	Health insurance	1.00								
	Medical aid	1.18 (1.16–1.21)	1.05 (1.05–1.05)	1.12 (1.04–1.21)	1.08 (1.04–1.12)	0.93 (0.86–1.01)	1.04 (0.93–1.16)	1.76 (1.65–1.87)	0.89 (0.74–1.06)	1.27 (1.14–1.41)
Type of healthcare facility	Tertiary/general hospital	1.00								
	Hospital	1.63 (1.59–1.68)	1.78 (1.78–1.79)	1.32 (1.18–1.47)	1.37 (1.31–1.43)	1.40 (1.18–1.65)	2.34 (1.94–2.82)	1.75 (1.61–1.89)	3.31 (2.77–3.96)	1.79 (1.56–2.05)
	Long-term care hospital	5.84 (5.56–6.13)	NA	5.58 (4.10–7.58)	7.23 (6.81–7.67)	12.6 (10.1–15.8)	13.7 (9.79–19.3)	2.18 (1.09–4.34)	1.25 (0.71–2.19)	3.06 (2.08–4.49)
	Psychiatric hospital	2.04 (1.95–2.13)	1.30 (1.30–1.31)	1.12 (0.95–1.33)	0.76 (0.61–0.95)	1.20 (0.63–2.26)	0.93 (0.66–1.33)	2.95 (2.72–3.19)	1.99 (1.53–2.60)	1.84 (1.56–2.17)
	Primary clinic	1.96 (1.92–1.99)	1.24 (1.24–1.24)	1.35 (1.28–1.43)	3.21 (3.09–3.33)	4.46 (4.14–4.81)	2.17 (1.93–2.43)	0.90 (0.82–0.98)	1.49 (1.34–1.65)	1.46 (1.33–1.60)
Department	Psychiatry	1.00								
	Neurology	0.72 (0.70–0.74)	1.26 (1.26–1.27)	0.85 (0.72–1.02)	0.98 (0.95–1.02)	0.84 (0.78–0.91)	0.66 (0.49–0.89)	1.44 (0.92–2.25)	0.89 (0.28–2.85)	1.09 (0.70–1.68)
	Internal medicine	1.68 (1.64–1.73)	1.77 (1.76–1.77)	1.64 (1.53–1.75)	2.06 (1.98–2.15)	1.10 (1.03–1.17)	1.24 (1.14–1.36)	2.24 (1.80–2.79)	0.90 (0.62–1.31)	2.68 (2.28–3.14)
	Other	1.50 (1.46–1.55)	4.37 (4.36–4.38)	1.69 (1.55–1.84)	1.58 (1.52–1.65)	1.21 (1.12–1.30)	1.36 (1.21–1.53)	0.89 (0.66–1.20)	2.49 (2.00–3.10)	2.69 (2.24–3.22)
Previous admission	No	1.00								
	Yes	1.06 (1.02–1.09)	1.06 (1.06–1.06)	1.09 (0.94–1.26)	0.85 (0.80–0.90)	1.41 (1.16–1.72)	1.14 (0.90–1.45)	1.18 (1.10–1.28)	0.90 (0.64–1.28)	1.03 (0.92–1.16)
Numbers of previous outpatient care	0	1.00								
	1–2	7.74 (7.51–7.97)	4.75 (4.75–4.76)	6.28 (5.84–6.75)	9.90 (9.25–10.6)	6.91 (6.46–7.40)	5.44 (4.94–5.99)	5.48 (4.06–7.42)	11.8 (9.5–14.6)	4.43 (3.57–5.49)
	3–5	11.1 (10.8–11.4)	7.32 (7.31–7.33)	9.44 (8.79–10.1)	13.0 (12.2–13.9)	7.86 (7.34–8.42)	7.15 (6.46–7.91)	12.1 (9.29–15.6)	24.4 (20.0–29.8)	5.88 (4.85–7.13)
	6–9	12.2 (11.8–12.5)	8.63 (8.62–8.64)	10.7 (9.94–11.5)	14.2 (13.3–15.1)	8.63 (7.97–9.33)	8.51 (7.67–9.43)	10.9 (8.4–14.1)	21.0 (17.2–25.7)	6.73 (5.59–8.11)
	≥ 10	12.5 (12.2–12.8)	9.19 (9.19–9.20)	10.2 (9.61–10.9)	14.7 (13.84–15.7)	7.51 (6.93–8.14)	9.15 (8.39–9.96)	15.4 (12.0–19.8)	20.2 (16.8–24.4)	6.43 (5.42–7.63)
CCI score	0	1.00								
	1	1.05 (1.02–1.07)	0.98 (0.98–0.99)	0.98 (0.93–1.03)	3.20 (2.81–3.66)	0.96 (0.90–1.02)	1.01 (0.94–1.09)	1.11 (1.04–1.19)	0.94 (0.84–1.05)	1.10 (0.99–1.21)
	2	1.06 (1.04–1.09)	1.07 (1.07–1.08)	0.93 (0.87–1.00)	3.35 (2.94–3.83)	0.94 (0.87–1.00)	1.00 (0.91–1.09)	1.01 (0.92–1.12)	0.82 (0.57–1.16)	1.12 (0.98–1.27)
	≥ 3	1.04 (1.01–1.06)	1.03 (1.03–1.03)	1.00 (0.94–1.07)	3.19 (2.80–3.64)	0.91 (0.85–0.97)	1.06 (0.97–1.16)	0.86 (0.77–0.96)	0.77 (0.41–1.43)	1.18 (1.03–1.35)
Diagnosis	Depression	1.00								
	Anxiety disorders	1.04 (1.01–1.06)								
	Dementia	2.60 (2.53–2.67)								
	Other mental disorders	2.36 (2.29–2.44)								
	Sleep disorders	1.15 (1.11–1.19)								
	Schizophrenia	1.40 (1.35–1.45)								
	BEDs	1.11 (1.05–1.17)								
	Bipolar disorder	1.38 (1.33–1.44)								
	Other	1.12 (1.09–1.15)								

Abbreviations: BED, behavioral and emotional disorders with onset usually in childhood and adolescence; CCI, Charlson comorbidity index; CI, confidence interval; NA, not applicable; OR, odds ratio; other mental disorders, other mental disorders due to brain damage and dysfunction and physical disease

## Discussion

The present study used nationwide health insurance claims data to investigate the utilization of telemedicine for mental disorders during the COVID-19 pandemic and compared the characteristics of patients who received telemedicine services with those of patients who received in-person therapies. We hypothesized that people with mild mental illness would be more likely to use telemedicine during the COVID-19 pandemic; however, the results of this study did not confirm our hypotheses.

It is essential to provide adequate explanation and obtain patient’s consent prior to telemedicine utilization [3]. We determined that severe mental illness may be more resistant to telemedicine than mild mental illness owing to lack of insight and functional decline [24]. However, in the special situation of the COVID-19 pandemic, it is estimated that the primary priority of telemedicine for patients with mental illness was more influenced by physicians and caregivers than by patient factors. A previous study reported that the outpatient visits for severe mental illness significantly declined as COVID-19 pandemic continued in Korea [25]. The prominence of telemedicine use for severe mental illness in our study may suggest that telemedicine may play a role in compensating for continuity of care during the pandemic period.

In this study, 2.1% of patients with depression, who received the most outpatient care, received telemedicine services, which was lesser than the corresponding proportions among those with dementia (6.7%), schizophrenia (4.4%), bipolar affective disorder (3.9%), and other mental disorders owing to brain damage, dysfunction, and physical disease (3.6%). Instead of people with mild mental health issues, older patients and patients with dementia were more likely to receive telemedicine services.

In Korea, telemedicine was estimated to be provided more to patients at high risk of infection or with difficulty moving. Based on this, it appears that telemedicine provided during the pandemic aimed to protect against the risk of disease and the preference of medical staff rather than to ensure accessibility and satisfy the desire of the patient.

The departments that treated mental illness were psychiatry, neurology, and internal medicine; among them, internal medicine used telemedicine treatment twice as much (2.7% vs. 2.9% vs. 6.4%, respectively). A previous study showed that telemedicine can reduce the number of secondary or tertiary illnesses by reducing the number of clinic visits in the older population with mental illness [18]. Patients with mental illness treated in internal medicine may have preferred telemedicine treatments owing to concerns regarding infection during the pandemic because they were relatively more likely to have physical diseases. This conforms with the principle that people with physical illnesses and visual or hearing impairments should be provided with the necessary medical services through telemedicine when isolated [26].

The proportion of patients receiving telemedicine services for mental illness was relatively low, at 2.3%, in tertiary or general hospitals (the highest was 22.6% in long-term care hospitals), followed by hospitals (4.5%), psychiatric hospitals (3.6%), and primary clinics (2.9%). The number of patients receiving telemedicine for mental illness was 47,363 at primary clinics, accounting for 59.1% of the total. In May 2020, an additional fee for telephone consultation management at the clinic level was applied to promote telemedicine, which increased the participation of primary medical institutions, and the results of the present study reflected this [27].

In this study, we found that women use telemedicine for mental illness more than men. A study on telemedicine utilization conducted in Korea during the COVID-19 pandemic showed a consistent tendency, of women using it more than men [28]. Studies in the United States conducted before the outbreak of COVID-19 have also reported that women preferred and used telemedicine more than men [29–31].

The study confirmed that patients hospitalized within the past year, those who visited outpatient clinics frequently, and those who had other underlying diseases were more likely to receive telemedicine treatment. However, patients with dementia and BED, with prior hospitalization, were less likely to receive telemedicine services. This means that the severity of physical illness in people with mental illness who received telemedicine treatment during the pandemic was higher than that in the general population. In general, people with mental illness have a lower life expectancy and worse physical health than those of the general population, reflecting the results of this study. [32].

Regarding disease-specific characteristics in severe mental illness, people with schizophrenia ranked third among patients with mental illnesses in the utilization of telemedicine. People with bipolar disorder ranked lower than those with depression, anxiety disorders, and sleep disorders in telemedicine use. The need for telemedicine may differ based on complex factors, such as the risk of infection, difficulty moving, severity of comorbidities, and the preference of physician and caregiver rather than based on the type of severe mental illness.

In a cohort study in the UK, the rate of refusal to receive the COVID-19 vaccine was the highest for people with schizophrenia, followed by those for patients with bipolar disorder and depression [33]. Restrictions on visits to medical institutions by unvaccinated people may be related to increased telemedicine use. The highest use by patients with dementia may be attributed to the concerns of caregivers of older adults, who are vulnerable to the risk of COVID-19 through visits to medical institutions [34, 35]. Additionally, restrictions on visitors or going out due to COVID-19 in long-term care facilities continued for a long time, probably resulting in telemedicine medical treatments from affiliated medical institutions among dementia patients. Previous studies on telemedicine for mental health during COVID-19 have included trauma and stressor-related disorders, anxiety disorders, depressive disorders, and eating disorders [20]. This result suggests that the interest in neurocognitive disorders, including dementia, will increase.

Unlike other countries, Korea responded to COVID-19 without imposing a national lockdown [36]. However, if people were not vaccinated against COVID-19, they could not enter medical institutions. Psychiatric hospitals and long-term care facilities minimized medical visits to outside medical institutions because of concerns regarding the spread of infection [36]. During the pandemic, telemedicine was used to fill the medical gaps of vulnerable populations. Concurrently, it was implemented in hospitalized patients with severe mental illness and dementia. This supports telemedicine as a useful tool in infectious disease situations for people with mental illness who have difficulty in accessing necessary services owing to chronic diseases.

During the COVID-19 pandemic, the trend in the number of telemedicine treatments received by patients with mental illnesses showed a pattern, in which the use of telemedicine increased in every wave period, when the number of confirmed patients rapidly increased, and then decreased. As the COVID-19 pandemic period was prolonged, the total numbers of telemedicine treatment tended to decrease. Korea had no hospitals closed because of COVID-19 and the use of telemedicine was very low compared with other countries during the pandemic [37]· [38]. Owing to low awareness of telemedicine among both healthcare providers and patients, it is believed that as the pandemic progresses, in-person therapies will be more preferred, with telemedicine remaining a secondary option [27]. Although the COVID-19 pandemic has removed some of the traditional barriers to telemedicine, there are still technological, infrastructural, educational, economic, and legal issues to consider to enable and sustain telemedicine [39–41].

### Limitations

This study had some limitations. First, the medical claims data did not have information on patient residence and, therefore, the impact of access to health care according to the region of residence could not be excluded. Second, the primary diagnosis was selected according to medical claim data, and mental illness has higher diagnostic accuracy than other diseases. Thus, coding issues for diagnosis may be relatively small [42]; however, as they are based on data for medical expense payment, incorrect diagnostic codes may be included in some cases. Third, as medical claims data include those who used medical institutions, vulnerable groups, such as refugees or residents of marginalized areas may be excluded, and there may be limitations in identifying digital equity. Fourth, during the COVID-19 pandemic, telemedicine increased access to medical care; however, health outcomes have not been analysed. Moreover, the safety of telemedicine in patients with severe conditions and older adults with many underlying physical diseases could not be examined. Further research is needed to examine this issue.

## Conclusions

In this study, people of older age and patients with dementia were more likely to use telemedicine during the COVID-19 pandemic. Additionally, the increase in the need of mental health treatment during the pandemic did not lead to telemedicine use. Therefore, based on these results, a policy for telemedicine in mental health is needed. In Korea, telemedicine is not fully authorized and was only allowed temporarily during the COVID-19 pandemic. After the end of the COVID-19 pandemic, telemedicine will settle into an essential role in future healthcare systems. To this end, the South Korean National Assembly and the government are attempting to gradually reform the medical law to permanently legalize telemedicine [43]. Telemedicine has been expanded and developed because of the COVID-19 pandemic; however, many improvements remain to be made to replace in-person care. In this process, it is important to consider policies and directions of telemedicine between countries [41]. In the face of repeated epidemics of infectious diseases, it is necessary to help maintain the continuity of telemedicine in various situations, including infectious disease outbreaks.

## Data Availability

The datasets during and/or analyzed during this study are available from the corresponding author and Health Insurance Review and Assessment Service (HIRA) upon reasonable request.

## Abbreviations

COVID-19: Coronavirus disease 2019
OR: Odds ratio
NHICD: National Health Insurance Claim Database
CCI: Charlson comorbidity index
BEDs: Behavioral and emotional disorders
CI: Confidence interval
